# Two new species of the leafhopper genus *Mitjaevia* Dworakowska from China (Hemiptera, Cicadellidae, Typhlocybinae)

**DOI:** 10.3897/BDJ.9.e72420

**Published:** 2021-10-08

**Authors:** Guimei Luo, Qingfa Song, Yuehua Song

**Affiliations:** 1 School of Karst Science, Guizhou Normal University / State Key Laboratory Cultivation Base for Guizhou Karst Mountain Ecology Environment of China, Guizhou, Guiyang 550001, China School of Karst Science, Guizhou Normal University / State Key Laboratory Cultivation Base for Guizhou Karst Mountain Ecology Environment of China, Guizhou Guiyang 550001 China

**Keywords:** homoptera, morphology, taxonomy, new taxa, karst

## Abstract

**Background:**

The leafhopper genus *Mitjaevia* Dworakowska,1970 includes 19 species worldwide, nine species are known in China and is widely distributed in Palaearctic and Oriental Regions

**New information:**

Two new species from Guizhou Province, China are described within the genus *Mitjaevia* Dworakowska, 1970 (Hemiptera: Cicadellidae: Typhlocybinae). A key to the species of the genus is provided and the female valvulae are described and figured.

## Introduction

The leafhopper genus *Mitjaevia* Dworakowska, 1970 was established in the tribe Erythroneurini of Typhlocybinae, with *Erythroneuraamseli* Dlabola, 1961 as its type species (Dworakowska 1970). The genus consists of 19 species worldwide, nine known species are from China (Chen and Song 2020) Table 1.

The characteristics of the leafhopper genus *Mitjaevia* are described as follows; dorsum yellow or dark brown and vertex usually with pair of dark spots. Pronotum pale or completely dark and distinctly wider than head. Eyes light brown or pale black. Above the dorsum of face, antennal foss with black spots. Abdomen apodemes small, narrow. Pronotum without conspicuous pits. Forewing outer apical cell about 2x as long as wide. Pcu vein on forewings distinct. Hind wing apex broadly rounded. Hind wing submarginal vein not extended to wing apex.

Male pygofer rounded or angled, with simple movably articulated dorsal appendage, not extended to apex of subgenital plate. Pygofer with setae on internal surface, with sparse long fine setae. Pygofer ventro-apical membranous area inconspicuous or absent directly. Subgenital plate long, curved dorsad, with many peg-like short rigid setae along upper margin from sub-base to middle part and three or more macrosetae present at mid-length. Subgenital plate section basad of medial constriction subequal to or shorter than distal section or longer than distal section. Style apex slender, pre-apical lobe usually well developed, large. Aedeagus with shaft tubular, sometimes with pair of processes. Connective "Y-" or "M-" shaped, with central lobe between lateral arms.

In this paper, two new species from Guizhou Province, China are described and illustrated. A key to all Chinese species of the genus *Mitjaevia* is given.

## Materials and methods

The leafhopper specimens were collected by sweep-net: collecting event once a month by an average of 100 nets each time (sweep net diameter of 50 cm) over grasslands. The collection time was in May with a temperature of 24℃, humidity of 25% and the altitude of 1541 m above sea level. The morphological terms used in this study follow Dietrich (2005) and Song and Li (2013). An Olympus SZX16 microscope was used for study materials and the Olympus BX53 microscope was used to draw the male genital anatomy. Overall habitus photos were taken using aKEYENCE VHX-5000 digital microscope. Body length as measured from the apex of vertex to the tip of forewing. All specimens examined are deposited in the collection of the School of Karst Science, Guizhou Normal University, China (GZNU).

## Taxon treatments

### Mitjaevia bifurcata sp. n.

B80A19C4-EE55-5C74-A21F-CC998EB741A0

C819273E-5613-4672-8E5B-47DCD50DA207

#### Materials

- **Type status:** Holotype. **Occurrence:** individualCount: 1; sex: male; lifeStage: adult; **Taxon:** scientificName: *Mitjaeviabifurcata*; order: Hemiptera; family: Cicadellidae; genus: Mitjaevia; specificEpithet: *bifurcata*; **Location:** country: China; stateProvince: Guizhou; locality: Bijie City, Qixinguan District, Salaxi Town; locationRemarks: label transliteration: "Guizhou, Bijie, 27.5.2019, coll. Zhouwei Yuan and Xiao Yang"; **Record Level:** collectionCode: Insects; basisOfRecord: Preserved Specimen
- **Type status:** Paratype. **Occurrence:** individualCount: 8; sex: 4 males, 4 females; lifeStage: adult; **Taxon:** scientificName: *Mitjaeviabifurcata*; order: Hemiptera; family: Cicadellidae; genus: Mitjaevia; specificEpithet: *bifurcata*; **Location:** country: China; stateProvince: Guizhou; locality: Bijie City, Qixinguan District, Salaxi Town;; locationRemarks: label transliteration: "Guizhou,Bijie, 27.5.2019, coll. Zhouwei Yuan and Xiao Yang"; **Record Level:** collectionCode: Insects; basisOfRecord: Preserved Specimen

#### Description

**Figure 1.**
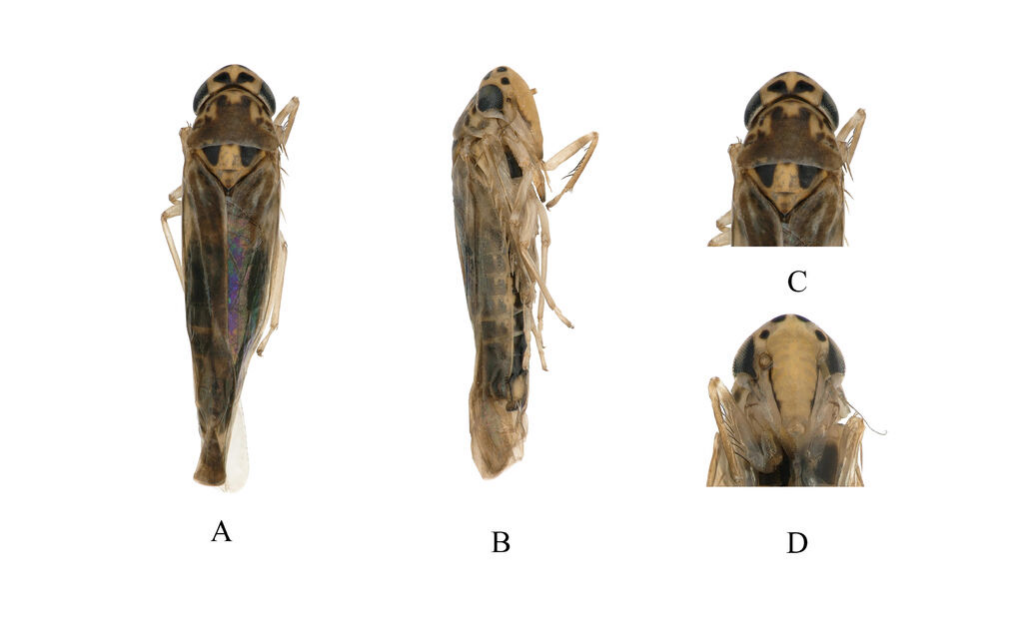
*Mitjaeviabifurcata* sp. n. **A.** Habitus, dorsal view **B.** Habitus, lateral view **C.** Head and thorax, dorsal view **D.** Face.

**Figure 2.**
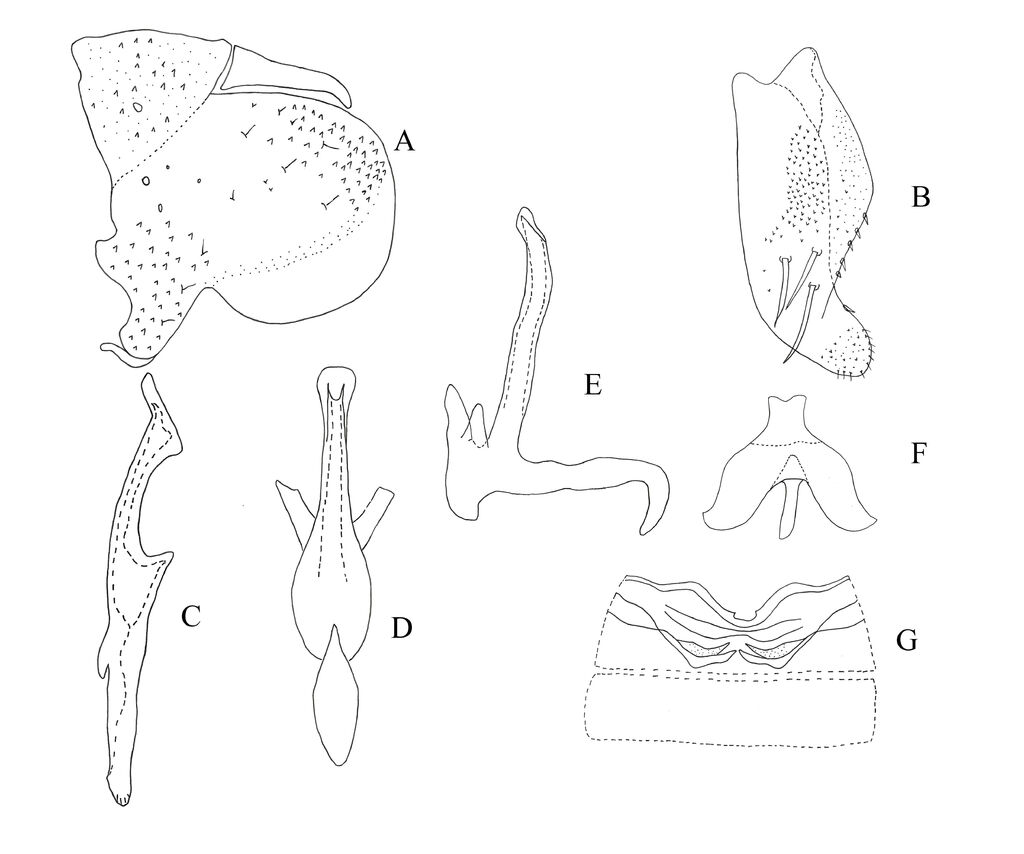
*Mitjaeviabifurcata* sp. n. **A.** Male pygofer, lateral view **B.** Subgenital plate **C.** Style **D.** Aedeagus, ventral view **E.** Aedeagus, lateral view **F.** Connective **G.** Abdominal apodemes.

Body length, males 2.9–3.0 mm, females 2.8–2.9 mm. Vertex (Fig. 1A) pale yellow, with pair of small black spots. Coronal suture short, with two irregular markings on sides (Fig. 1A and C). Eyes greyish-black. Pronotum yellow, with irregularly dark brown or black patches (Fig. 1A and C). Scutellum light yellow, with basal triangles black and transverse impression distinct (Fig. 1A and C). Face light brownish-yellow, frontoclypeus with black patches at sides basally (Fig. 1D). Forewing milky white, with light brown patches. Abdominal apodemes small, not extended to hind margin of 3^rd^ sternite (Fig. 2G).

#### Diagnosis

**Male genitalia.** Pygofer lobe broad, with many microtrichia and fine setae scattered on lateral surface, occasionally with long fine setae. Pygofer dorsal appendage expanded basally, caudal margin round (Fig. 2A). Subgenital plate short, with three long macrosetae on lateral surface and row of peg-like setae along dorsal margin to medium area, with sparse fine small setae on apical portion (Fig. 2B). Style long, with pre-apical lobe moderately large (Fig. 2C). Preatrium of aedeagus short and aedeagus shaft slender; at the base of aedeagus with small appendages ; dorsal apodeme branched apically; gonopore apical on ventral surface (Fig. 2D and E). Connective Y-shaped, arms and stem developed, with long and thin central lobe (Fig. 2F).

**Figure 3.**
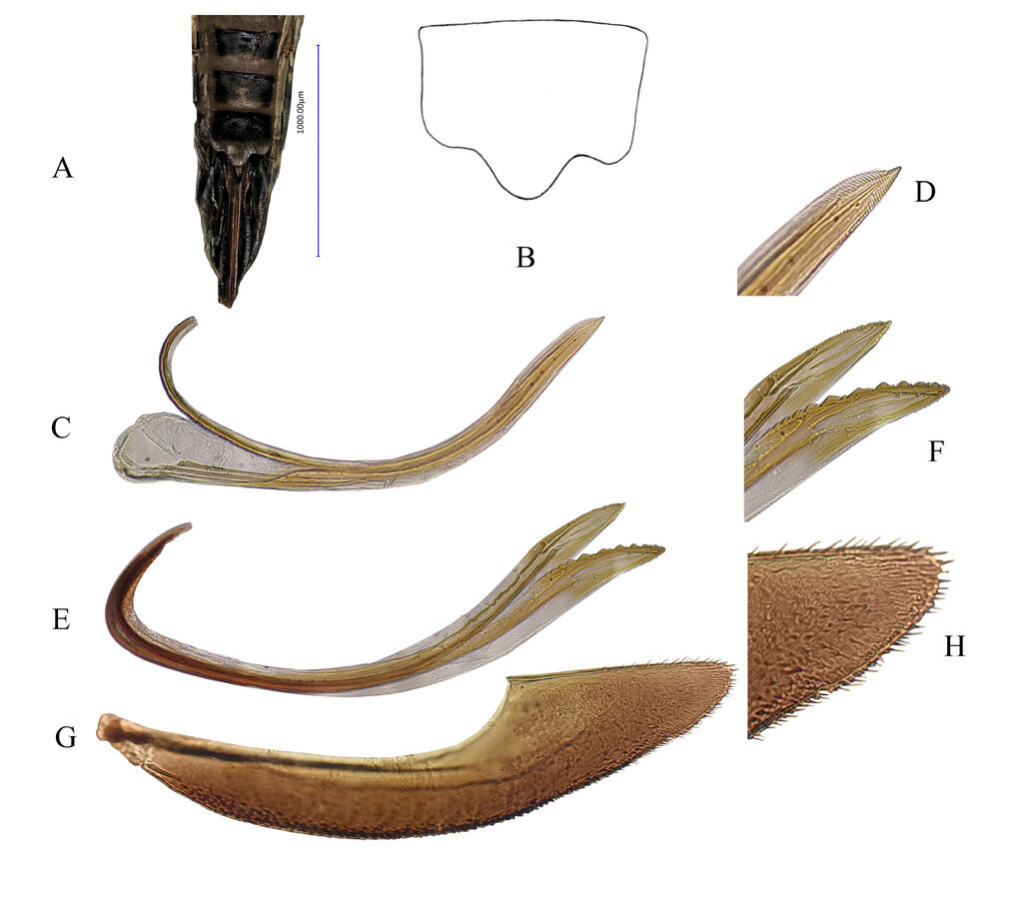
*Mitjaeviabifurcata* sp. n. **A.** Female pygofer **B.** Sternite VII **C.** Valvula I **D.** Partial enlargement of valvula I **E.** Valvulae II **F.** Partial enlargement of valvulae II **G.** Valvula III **H.** Partial enlargement of valvula III.

**Female genitalia.** Female 7^th^ sternite as in Fig. 3A and B. Valvula I elongate, curved dorsad, apical portion pointed, with dense imbricate carving (Fig. 3C and D). Valvulae II elongate, gradually expanded from base to top and divided into two blades, one of which has dentate protrusions (Fig. 3E and F). Valvula III leaf-like, with microtrichia on dorsal margin and apex (Fig. 3G and H).

#### Etymology

The new species is named from the Latin word “bifurcatus”, referring to dorsal apodeme branched of the aedeagus.

#### Taxon discussion

This species has a similar aedeagus shape to *Mitjaeviaprotuberanta* Song, Li, Xiong, 2011 (Song et al. 2011), but it can be distinguished by the aedeagal shaft slender; pre-atrium of aedeagus short, dorsal apodeme branched apically and without triangle-like processes subapically.

**Distribution.** Guizhou Province.

### Mitjaevia ramosa sp. n.

499DCE4F-4902-5EDC-913E-843B2F442F61

E0DEFC13-83FF-4318-B067-1680826280D7

#### Materials

- **Type status:** Holotype. **Occurrence:** individualCount: 1; sex: male; lifeStage: adult; **Taxon:** scientificName: *Mitjaeviaramosa*; order: Hemiptera; family: Cicadellidae; genus: Mitjaevia; specificEpithet: *ramosa*; **Location:** country: China; stateProvince: Guizhou; county: Huajiang; locationRemarks: label transliteration: "Guizhou Huajiang, 23. 5. 2019, coll. Zhouwei Yuan and Xiao Yang"; **Record Level:** collectionCode: Insects; basisOfRecord: Preserved Specimen
- **Type status:** Paratype. **Occurrence:** individualCount: 1; sex: male; lifeStage: adult; **Taxon:** scientificName: *Mitjaeviaramosa*; order: Hemiptera; family: Cicadellidae; genus: Mitjaevia; specificEpithet: *ramosa*; **Location:** country: China; stateProvince: Guizhou; county: Huajiang; locationRemarks: label transliteration: "Guizhou Huajiang, 23. 5. 2019, coll. Zhouwei Yuan and Xiao Yang"; **Record Level:** collectionCode: Insects; basisOfRecord: Preserved Specimen

#### Description

**Figure 4.**
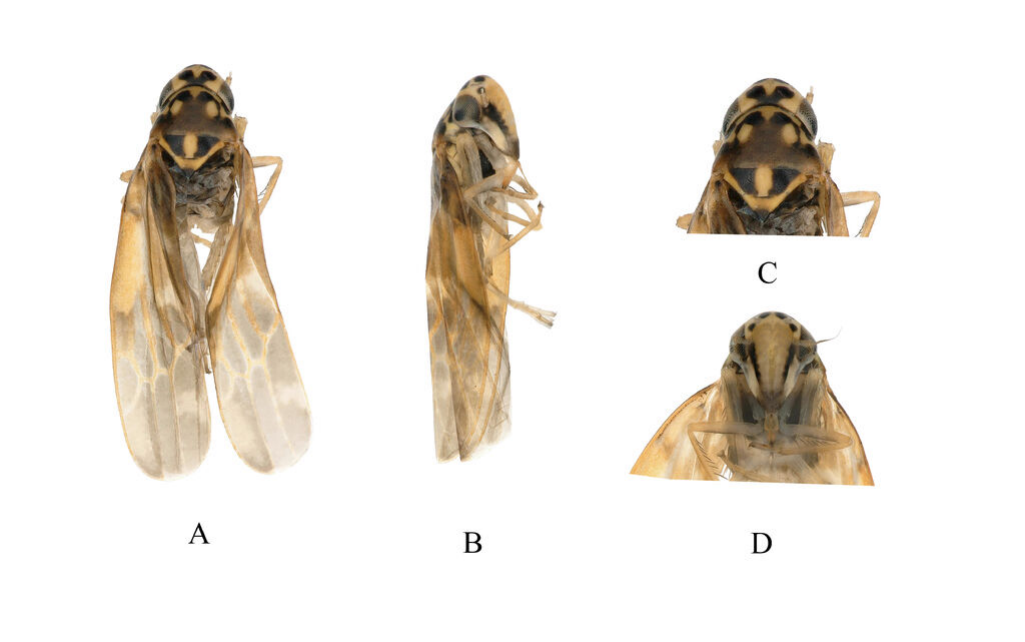
*Mitjaeviaramosa* sp. n. **A.** Habitus, dorsal view **B.** Habitus, lateral view **C.** Head and thorax, dorsal view **D.** Face.

**Figure 5.**
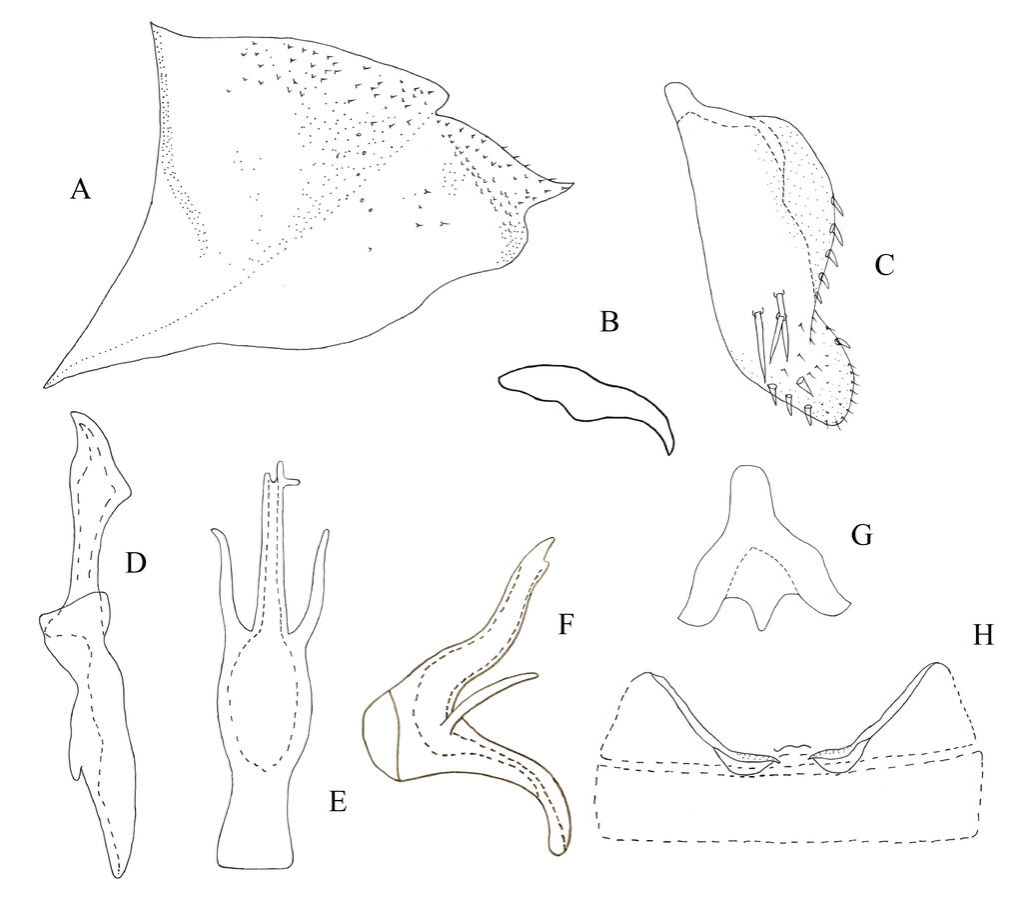
*Mitjaeviaramosa* sp. n. **A.** Male pygofer, lateral view **B.** Pygofer dorsal appendage, lateral view **C.** Subgenital plate **D.** Style **E.** Aedeagus, ventral view **F.** Aedeagus, lateral view **G.** Connective **H.** Abdominal apodemes.

Body length, males 2.50-2.70 mm. Vertex (Fig. 4A) light yellow, with pair of small black spots. Coronal suture short, with two irregular black markings on both sides (Fig. 4A and C). Eyes greyish-black. Pronotum yellowish, with symmetrical pale-yellow oval impressed patches medially, (Fig. 4A and C). Scutellum yellow, with black lateral triangles, transverse impression distinct. Face light brownish-yellow, frontoclypeus with black patches at sides basally; anteclypeus dark brown (Fig. 4D). Forewing with orange and grey patches. Abdominal apodemes small, extended to hind margin of 3^rd^ sternite (Fig. 5H).

#### Diagnosis

**Male genitalia.** Pygofer lobe broad, with numerous microtrichia and fine setae scattered near caudal part and dorsal margin. Pygofer dorsal appendage expanded basally, tapering to apex and hook-like apically (Fig. 5A and B). Subgenital plate short, wide and midfield slightly concave, with three macrosetae, numerous peg-like setae along dorsal margin (Fig. 5C). Style apex expanded, "curved neck" area slender, pre-apical lobe obvious, enlarged (Fig. 5D). Pre-atrium of aedeagus little expanded in lateral view, aedeagus shaft slender, with pair of "finger-like" processes arising from base of shaft and extending outwards, bifurcated into two branches apically (Fig. 5E and F). Connective Y-shaped, two lateral arms and stem developed, median anterior lobe well developed (Fig. 5G).

#### Etymology

The new species is named from the Latin word “ramosus”, referring to the aedeagal shaft with two bifurcated branches at apex.

#### Taxon discussion

The new species is similar to *Mitjaeviadiana* (Distant 1918), but differs in having the "finger-like" processes arising from base of aedeagal shaft and extending outwards; two bifurcated branches at apex; connective Y-shaped and stem developed.

**Distribution.** Guizhou Province.

## Identification Keys

### Key to species of *Mitjaevia* from China (males)

**Table:** 

1	Aedeagus with one or two pairs of processes	6
–	Aedeagus without process	2
2	Scutellum with one pair of small dark dots above transverse impression	*M.korolevskayae*
–	Scutellum without small dark dots above transverse impression	3
3	Aedeagal shaft cylindrical, evenly tapered from base to apex	4
–	Aedeagal shaft laterally compressed, abruptly tapered from subapically to apex	5
4	Pre-atrium of aedeagus long in lateral view (Fig. 7b)	*M.nanaoensis*
–	Pre-atrium of aedeagus short in lateral view (Fig. 7c)	*M.tappana*
5	Style with pre-apical lobe small; aedeagal shaft with rounded apex in lateral view (Fig. 7d)	*M.shibingensis*
–	Style with pre-apical lobe large; aedeagal shaft with acute apex in lateral view (Fig. 7e, f)	*M.dworakowskae*
6	Aedeagus with two pairs of processes	7
–	Aedeagus with one pair of processes	10
7	Aedeagal shaft bifurcate at apex	8
–	Aedeagal shaft not bifurcate at apex	9
8	Aedeagal shaft with four apical branches at apex, with pair of thin, sickle-like processes (Fig. 6c)	*M.diana*
–	Aedeagal shaft with two round branches at apex and one pair of finger-like processes at base (Fig. 5)	*M.ramosa* **sp. n.**
9	Aedeagus shaft with pair of small, triangle-like processes subapically (Fig. 6b)	*M.protuberanta*
–	Aedeagal shaft with apex extended, without process (Fig. 2)	*M.bifurcata* **sp. n.**
10	Aedeagal shaft with paired processes basally (Fig. 7a)	*M.aurantiaca*
–	Aedeagal shaft with paired processes subapically (Fig. 6a)	*M.wangwushana*

## Supplementary Material

XML Treatment for Mitjaevia bifurcata

XML Treatment for Mitjaevia ramosa

## Figures and Tables

**Figure 6a.**
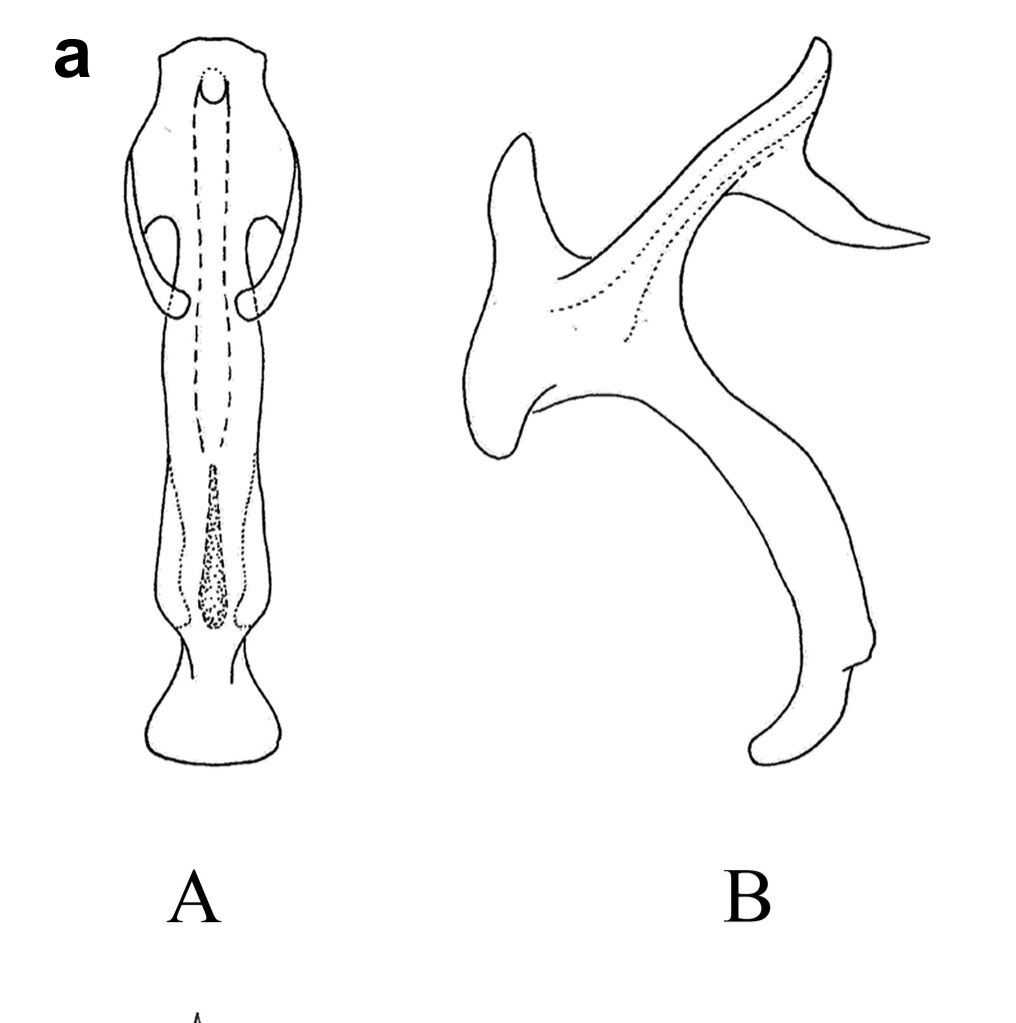
*M.wangwushana* Song, Li & Xiong **A.** aedeagus, ventral view **B.** aedeagus, lateral view

**Figure 6b.**
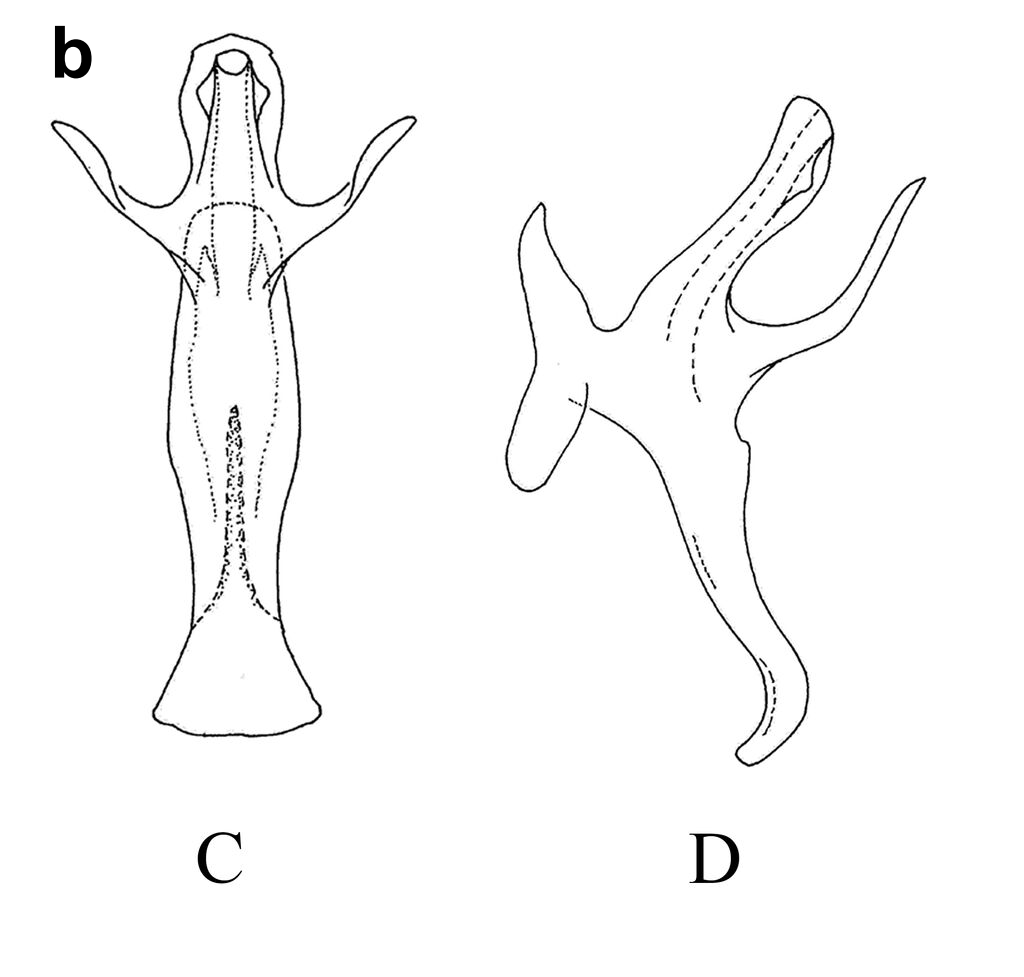
*M.protuberanta* Song, Li & Xiong **C.** aedeagus, ventral view **D**. aedeagus, lateral view

**Figure 6c.**
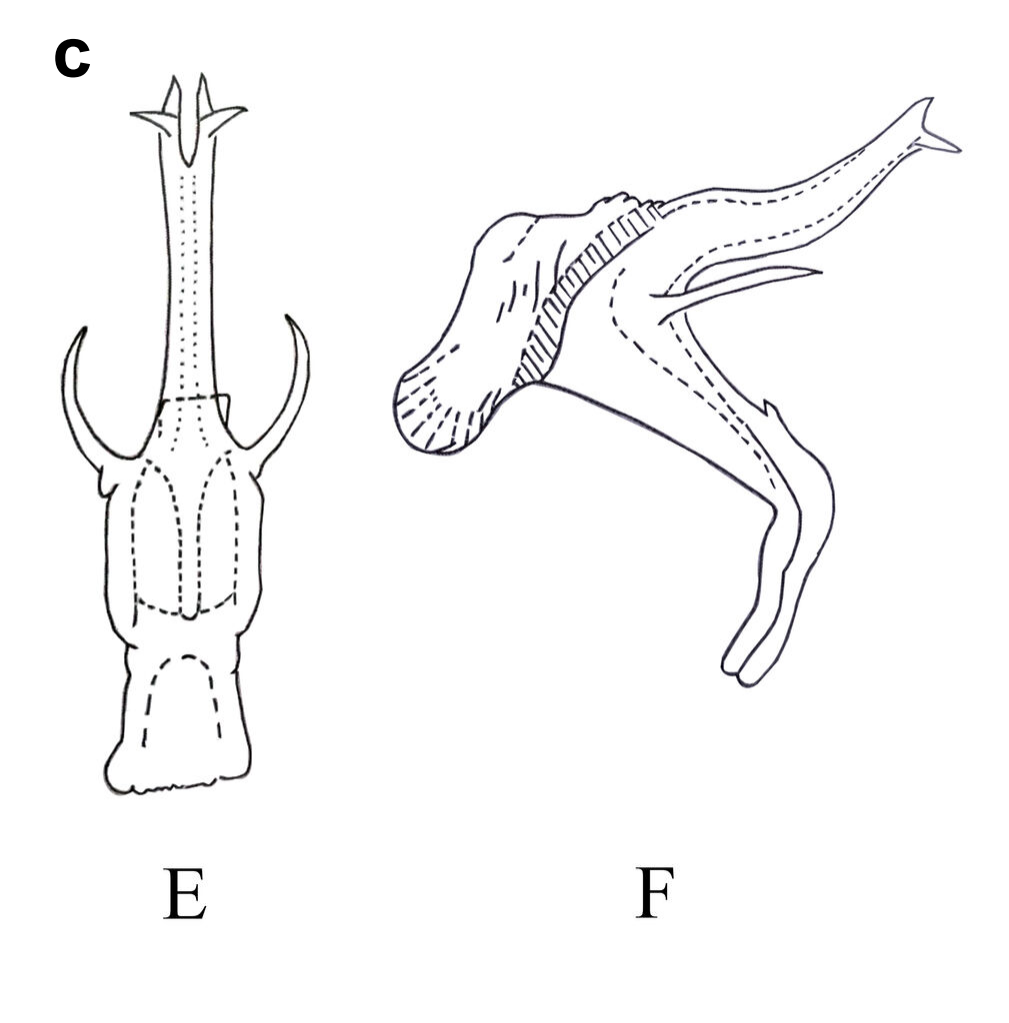
*M.diana* Distant **E.** aedeagus, ventral view **F.** aedeagus, lateral view

**Figure 6d.**
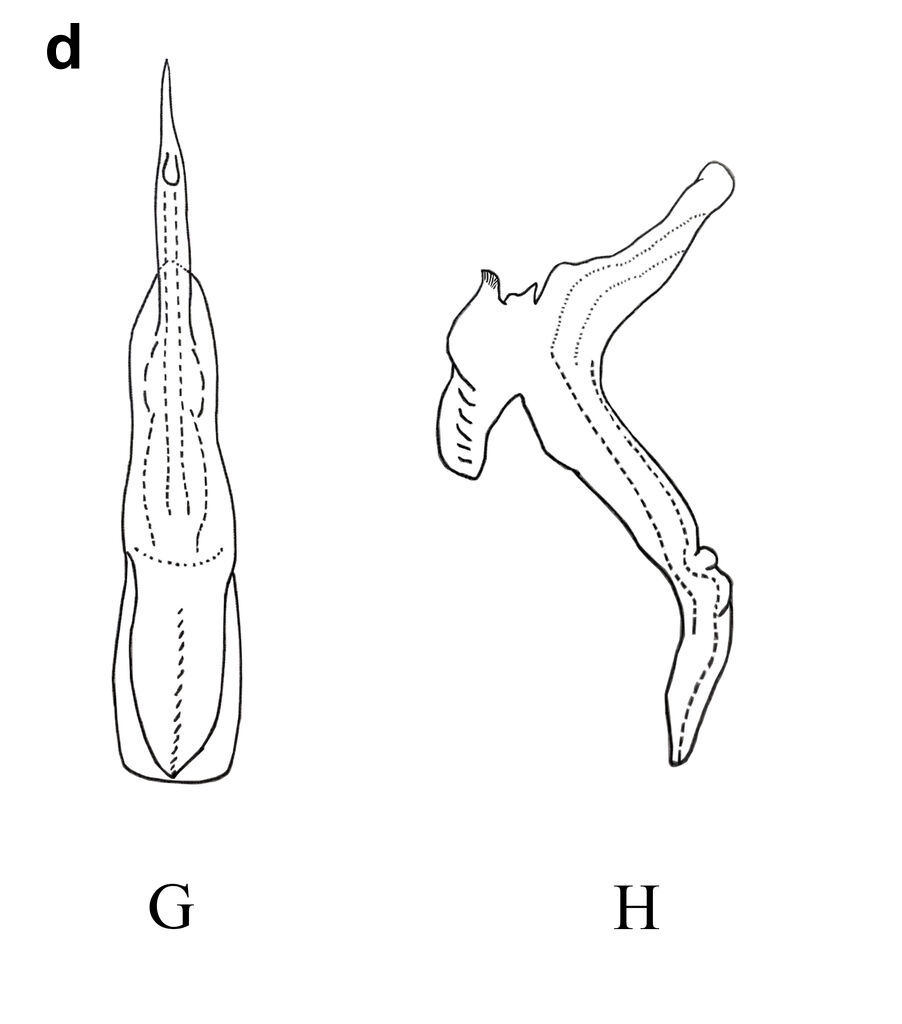
*M.korolevskayae* Dworakowska **G.** aedeagus, ventral view **H.** aedeagus, lateral view.

**Figure 7a.**
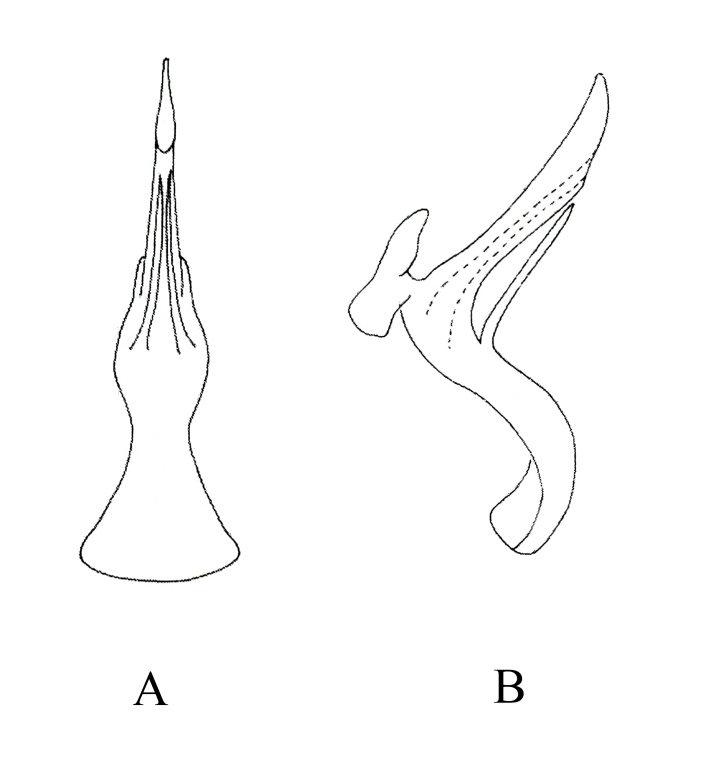
*M.aurantiaca* Mitjaev **A.** aedeagus, ventral view **B.** aedeagus, lateral view

**Figure 7b.**
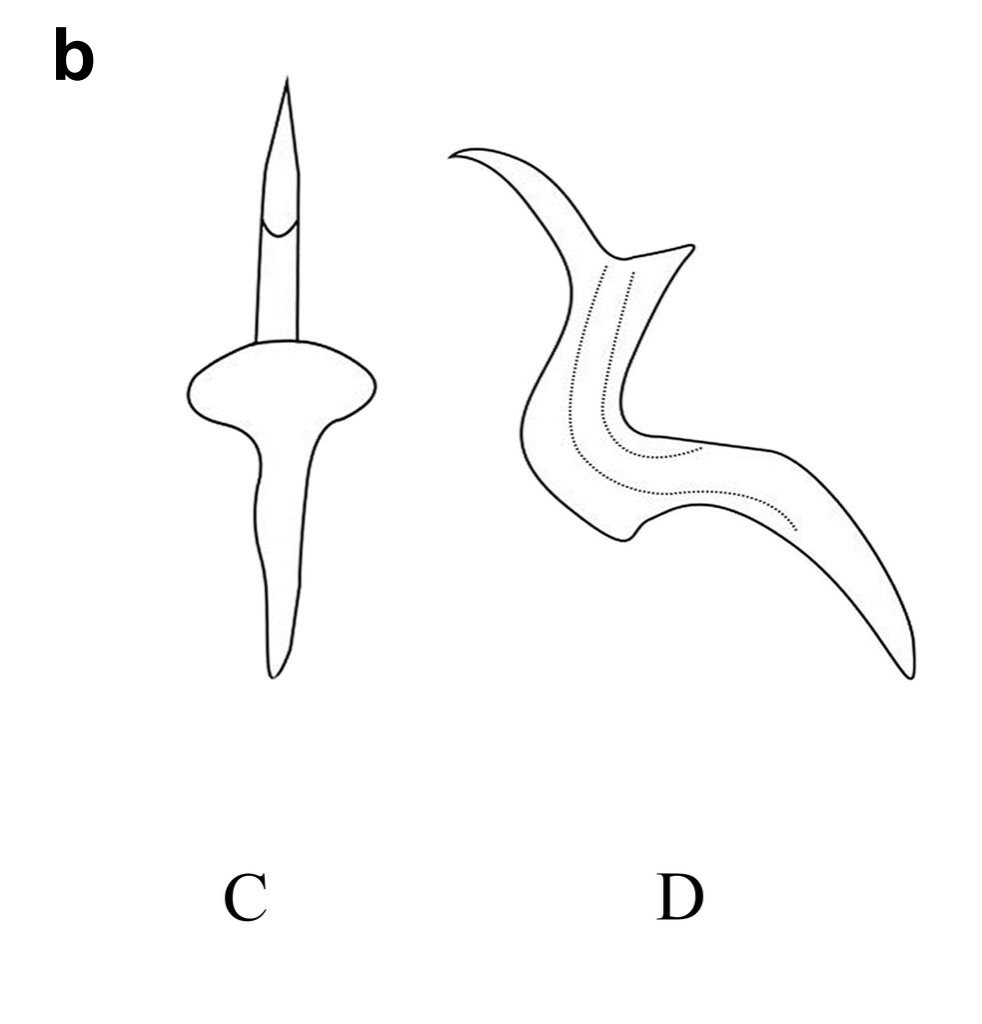
*M.nanaoensis* Chiang & Knight **C.** aedeagus, ventral view **D.** aedeagus, lateral view

**Figure 7c.**
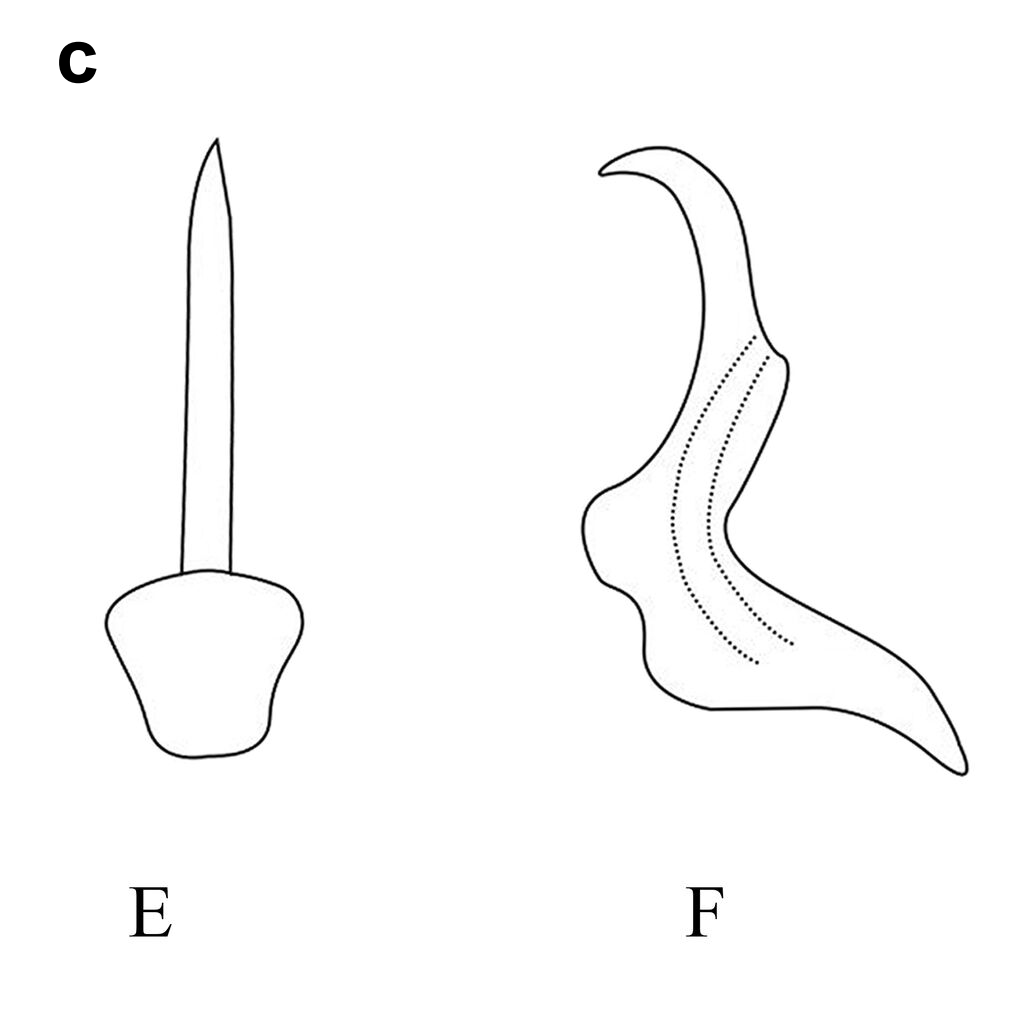
*M.tappana* Chiang & Knight **E.** aedeagus, ventral view **F.** aedeagus, lateral view

**Figure 7d.**
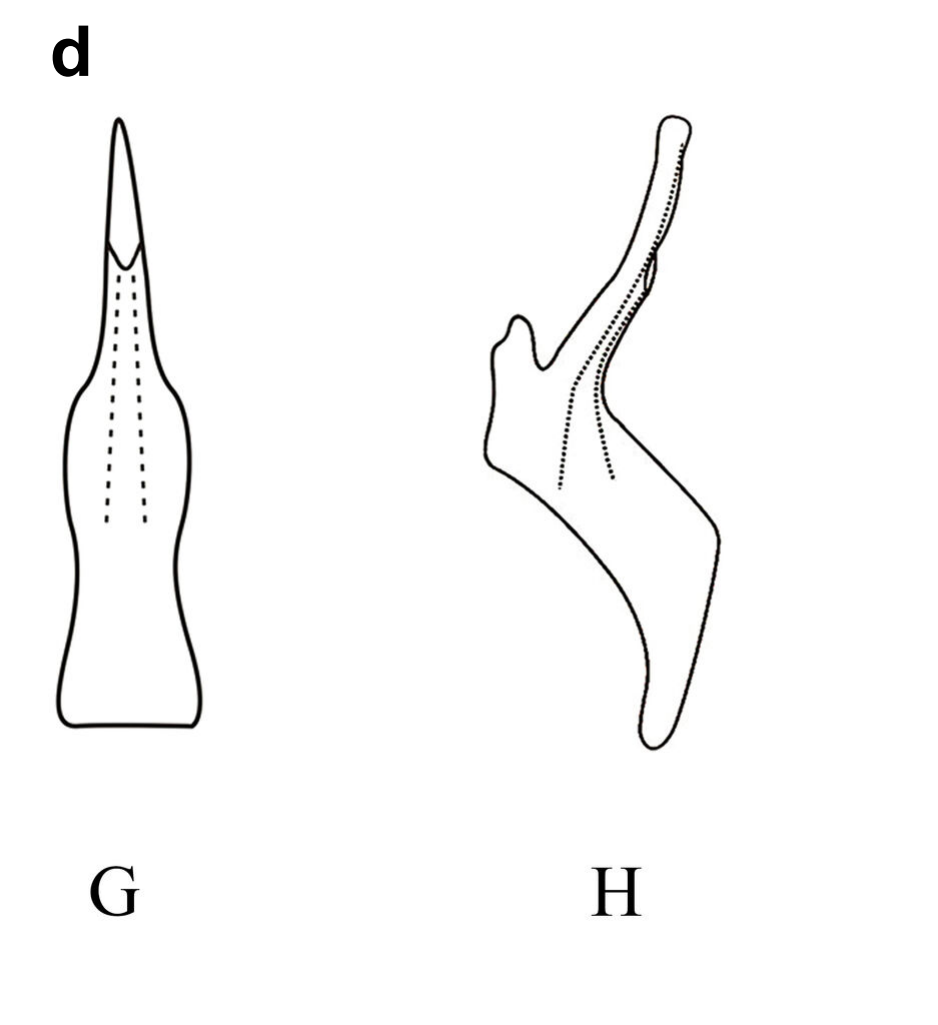
*M.shibingensis* Chen, Song **G.** aedeagus, ventral view **H.** aedeagus, lateral view

**Figure 7e.**
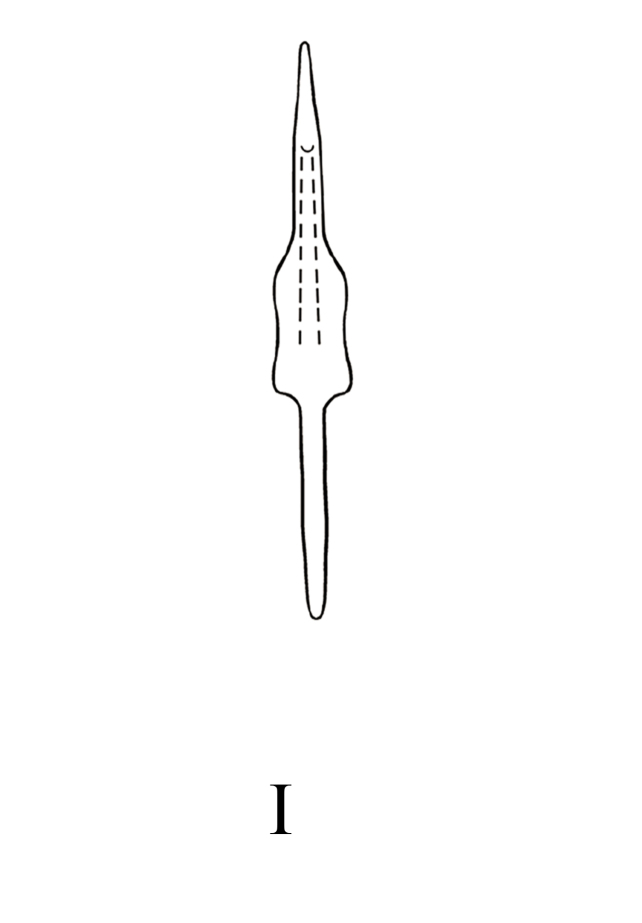
*M.dworakowskae* Chen, Song **I.** aedeagus, ventral view

**Figure 7f.**
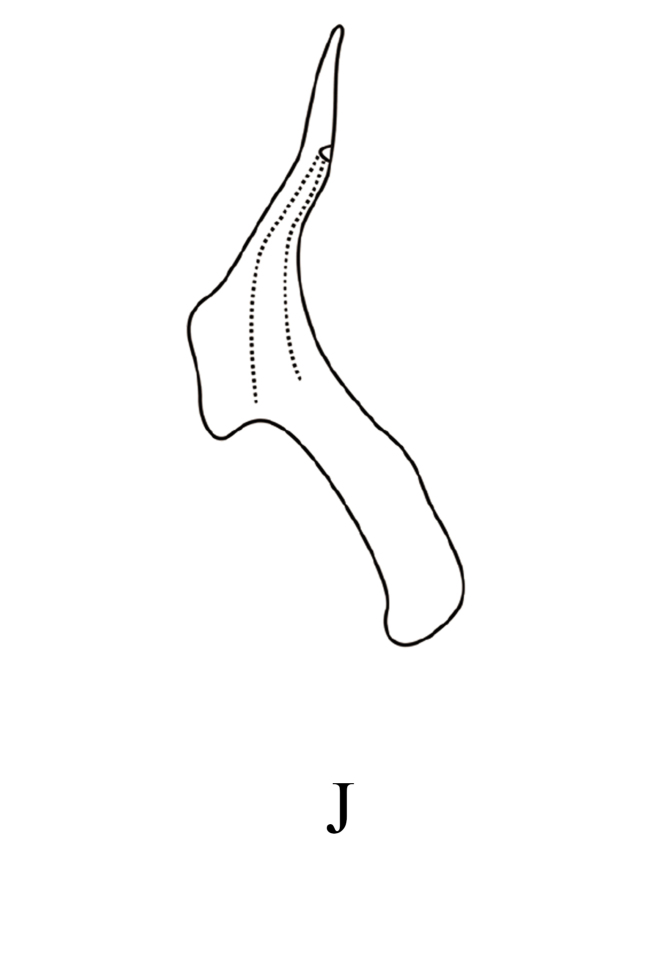
*M.dworakowskae* Chen, Song **J.** aedeagus, lateral view.

**Table 1.** *Mitjaevia* species distribution table

Species name	Distribution
*M.amseli*	Kyrgyzstan, Uzbekistan , Afghanistan, Altai Mts, Russia, Kazakhstan, Kyrgyzstan, Tadzhikistan, Tajikistan, Uzbekistan
*M.atropictila*	India, Pakistan
*M.aurantiaca*	Kazakhstan, Tadzhikistan, China
*M.aurea*	India
*M.bibichanae*	Kazakhstan, Tadzhikistan, Uzbekistan
*M.callosa*	India
*M.diana*	India, Kazakhstan China
*M.elegantula*	India
*M.korolevskayae*	Vietnam, China
*M.maculata*	India, Pakistan
*M.nanaoensis*	Taiwan, China
*M.narzikulovi*	Tadzhikistan
*M.notata*	Bangladesh, India
*M.protuberanta*	China
*M.sikkimensis*	India
*M.tappana*	China
*M.wangwushana*	China
*M.shibingensis*	China
*M.dworakowskae*	China
